# Ertugliflozin and incident obstructive sleep apnea: an analysis from the VERTIS CV trial

**DOI:** 10.1007/s11325-022-02594-2

**Published:** 2022-05-20

**Authors:** Brian S. Wojeck, Silvio E. Inzucchi, Ian J. Neeland, James P. Mancuso, Robert Frederich, Urszula Masiukiewicz, Nilo B. Cater, Darren K. McGuire, Christopher P. Cannon, Henry Klar Yaggi

**Affiliations:** 1 Section of Endocrinology, Yale School of Medicine, 33 Cedar Street, P.O. Box 208020, New Haven, CT 06520 USA; 2 Harrington Heart and Vascular Institute University Hospitals Cleveland Medical Center and Case Western Reserve University School of Medicine, Cleveland, OH USA; 3 Pfizer Inc, Groton, CT USA; 4 Pfizer Inc, Collegeville, PA USA; 5 Pfizer Inc, New York, NY USA; 6 University of Texas Southwestern Medical Center and Parkland Health and Hospital System, Dallas, TX USA; 7 Cardiovascular Division, Brigham and Women’s Hospital, Harvard Medical School, Boston, MA USA; 8 Section of Pulmonary, Critical Care and Sleep Medicine, Yale School of Medicine, New Haven, CT USA; 9 The VA CT Clinical Epidemiology Research Center, West Haven, CT USA

**Keywords:** Sodium glucose transporter type 2 inhibitor, SGLT2, Sleep apnea, OSA, Sleep disordered breathing

## Abstract

**Purpose:**

The sodium-glucose transporter 2 inhibitor (SGLT2i) empagliflozin may reduce the incidence of obstructive sleep apnea (OSA) in patients with type 2 diabetes (T2D) and cardiovascular (CV) disease. This analysis of VERTIS CV, the CV outcome trial for the SGLT2i ertugliflozin conducted in a similar group of patients, explored the effects of ertugliflozin on reported incident OSA.

**Methods:**

In VERTIS CV, patients ≥ 40 years with T2D and atherosclerotic CV disease (ASCVD) were randomized to ertugliflozin 5 or 15 mg or placebo. The primary endpoint was the composite of major adverse CV events. This exploratory analysis evaluated the impact of ertugliflozin (5 and 15 mg pooled) on incident OSA. Patients with prevalent OSA were excluded. Incident OSA events were based on investigator-reported events using the MedDRA SMQ term “sleep apnea syndrome.” A multivariable Cox proportional hazards regression model was constructed to assess the association between ertugliflozin and incident OSA.

**Results:**

Of 8246 patients enrolled, 7697 (93.3%) were without baseline OSA (placebo, *n* = 2561; ertugliflozin, *n* = 5136; mean age 64.4 years; BMI 31.7 kg/m^2^; HbA1c, 8.2%; 69.2% male; 88.3% White). The OSA incidence rate was 1.44 per 1000 person-years versus 2.61 per 1000 person-years among patients treated with ertugliflozin versus placebo, respectively, corresponding to a 48% relative risk reduction (HR 0.52; 95% CI 0.28–0.96; *P* = 0.04).

**Conclusions:**

In VERTIS CV, ertugliflozin reduced by nearly half the incidence of OSA in patients with T2D and ASCVD. These data contribute to the literature that SGLT2is may have a significant beneficial impact on OSA.

Trial registration.

ClinicalTrials.gov identifier: NCT01986881.

## Introduction

Obstructive sleep apnea (OSA) has been independently associated with important health outcomes, including hypertension, cardiovascular (CV) events, stroke, heart failure, chronic kidney disease (CKD), and metabolic dysfunction (1–3). OSA prevalence in adults ranges between 9 and 36% with higher rates in older men [4]. Among individuals with type 2 diabetes (T2D), concomitant OSA is associated with a higher risk of CV disease (CVD), atrial fibrillation, peripheral neuropathy, diabetic foot disease, CKD, and all-cause mortality [3]. Furthermore, in a randomly selected, nationally representative sample of Medicare beneficiaries, patients with untreated OSA had significantly higher mean total annual healthcare costs [5]. Treatment adherence with continuous positive airway pressure (CPAP), the mainstay intervention for OSA, is poor, ranging from 34 to 60% [6]. While improving CPAP adherence is key to improving OSA outcomes, the discovery of effective medical therapies could transform OSA care.

The sodium-glucose cotransporter 2 inhibitors (SGLT2i) were initially developed and approved to treat hyperglycemia in patients with T2D, but potent CV and kidney benefits were subsequently demonstrated in large-scale randomized trials. Their potential efficacy in OSA is just beginning to be explored. In a recent study, 36 patients with T2D and OSA were randomized to either metformin and glimepiride or metformin and the SGLT2i dapagliflozin and treated for 24 weeks. Home sleep testing was performed at the beginning and end of the study. Dapagliflozin led to significantly improved apnea–hypopnea index (AHI), a small rise in oxygen saturation, and better scores on the Epworth Sleepiness Scale [7]. In another small retrospective study of patients with OSA who had refused CPAP therapy, SGLT2i treatment was associated with significantly lower AHI [8]. Results from analyses exploring the effect of empagliflozin on OSA incidence from the EMPA-REG OUTCOME trial found that empagliflozin versus placebo reduced the incidence of OSA in patients with T2D and atherosclerotic CVD (ASCVD) by more than half (4.6/1000 patient-years [1.13%] vs. 2.2/1000 patient-years [0.57%]; hazard ratio [HR], 0.48; 95% confidence interval [CI], 0.27–0.83; *P* = 0.009) (9). Notably, this effect was largely independent of weight loss.

To further investigate the effect of SGLT2 inhibition on OSA incidence, we performed a post hoc analysis of VERTIS CV, a double-blind, placebo-controlled trial of the efficacy and safety of the SGLT2i ertugliflozin in patients with T2D and ASCVD.

## Method

VERTIS CV (NCT01986881) consisted of 8246 patients ≥ 40 years of age with T2D and ASCVD randomized to ertugliflozin 5 mg, 15 mg, or placebo once daily, with the primary outcome of composite of major adverse CV events. In these exploratory analyses, we evaluated the impact of ertugliflozin on incident OSA. A pooled analysis of ertugliflozin 5 and 15 mg (measured from the first dose of study medication until treatment end plus an additional 14 days) versus placebo was conducted. Patients with OSA at baseline (549; 6.7%) were excluded. Incident OSA events were based on investigator-reported adverse events during the trial using the MedDRA term sleep apnea syndrome (synonyms at: https://bioportal.bioontology.org/ontologies/MEDDRA?p=classes&conceptid=10040979). Kaplan–Meier estimates for time to first OSA event by treatment assignment for participants without OSA at baseline were plotted. A stratified Cox proportional hazards regression model was constructed to compare treatments with adjustment for age, sex, geographic region, baseline body mass index (BMI; < 35, ≥ 35 kg/m^2^), baseline glycated hemoglobin (HbA1c; < 9.0, ≥ 9.0%), and baseline estimated glomerular filtration rate (eGFR; < 60, ≥ 60 mL/min/1.73 m^2^) and with stratification by enrollment cohort (before and after protocol amendment).

## Results

**Figure 1:**
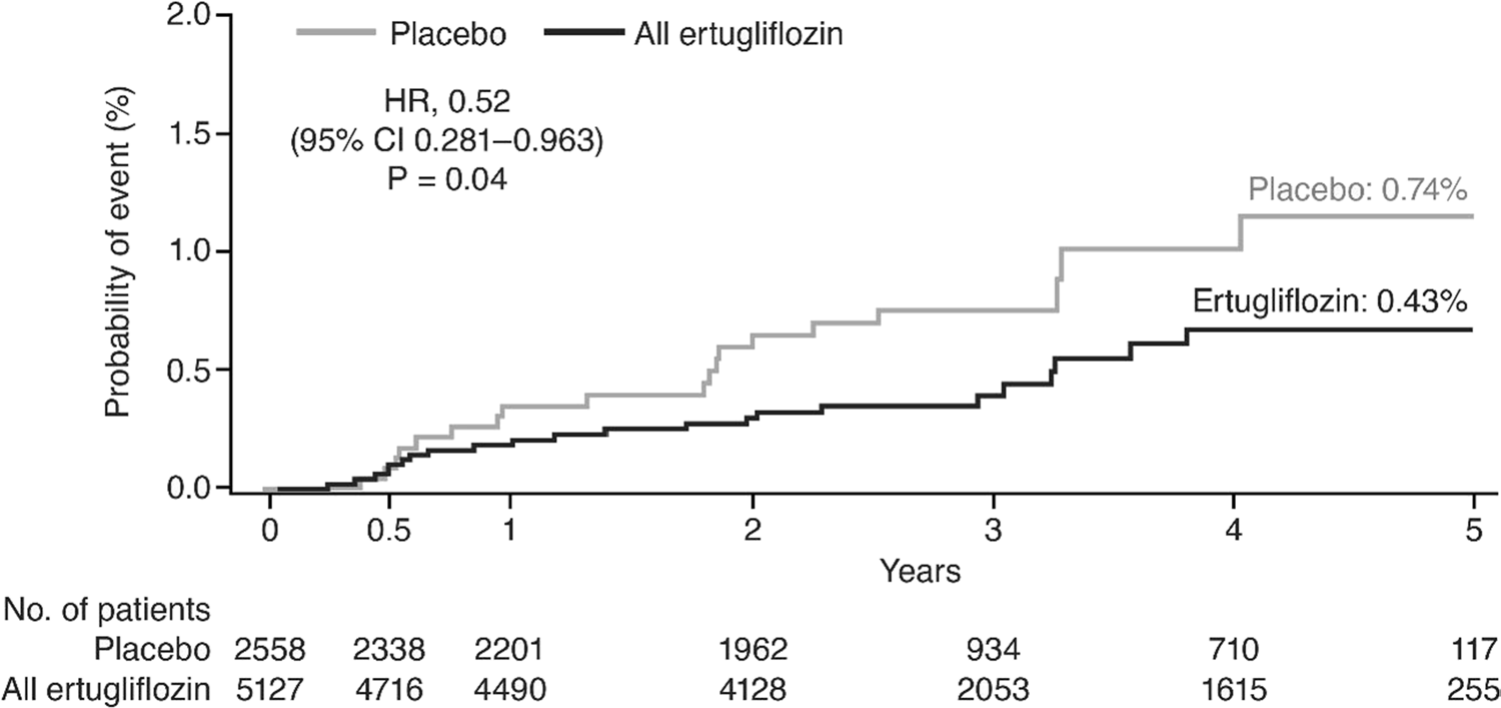
Fig. 1 Kaplan–Meier estimates for time to first OSA event on treatment for patients without OSA at baseline. OSA events are based on adverse events that occurred between the date of the first dose of study medication until treatment end plus an additional 14 days. HR, 95% CI, and *P* value are based on a stratified Cox proportional hazards model that includes categorical terms for treatment, sex, geographical region, baseline BMI, baseline HbA1c, and baseline eGFR, a continuous term for age, and a stratification factor for enrollment cohort. *BMI* body mass index; *CI* confidence interval; *eGFR* estimated glomerular filtration rate; *HbA1c* glycated hemoglobin; *HR* hazard ratio; *OSA* obstructive sleep apnea

In VERTIS CV, 7697 (93.3%) patients were without baseline OSA. These patients were primarily White (88.3%), male (69.2%), with mean age of 64.3 years, BMI 31.7 kg/m^2^, HbA1c 8.2% and eGFR 76.2 mL/min/1.73 m^2^. Among those without baseline OSA, the event rate for incident OSA with ertugliflozin was 1.4 per 1000 patient-years compared with 2.6 per 1000 patient-years with placebo (Table 1), corresponding to a 48% relative risk reduction for incident OSA in patients on ertugliflozin versus placebo (HR 0.52; 95% CI 0.28–0.96; *P* = 0.04). There was an early differentiation in the incidence of OSA with ertugliflozin versus placebo (Fig. 1).

**Table 1:** Time to first OSA event for patients without OSA at baseline

	All ertugliflozin (*N* = 5126)		Placebo (*N* = 2557)		HR* (95% CI)
	No. (%)	Rate/1000 patient- years	No. (%)	Rate/1000 patient- years	
OSA event	22 (0.43)	1.4	19 (0.74)	2.6	0.52 (0.28–0.96); *P* = 0.04

OSA events are based on adverse events that occurred between the date of the first dose of study medication until treatment end plus an additional 14 days

^*^Ertugliflozin (pooled) vs. placebo, based on the stratified Cox proportional hazards model that includes categorical terms for treatment, sex, geographical region, baseline BMI, baseline HbA1c, and baseline eGFR, a continuous term for age, and a stratification factor for enrollment cohort

*BMI* body mass index, *CI* confidence interval, *eGFR* estimated glomerular filtration rate, *HbA1c* glycated hemoglobin, *HR* hazard ratio, *OSA* obstructive sleep apnea

## Discussion

Small studies have suggested the potential efficacy of SGLT2i versus placebo in OSA (7, 8). An exploratory analysis of the large EMPA-REG OUTCOME trial suggested that empagliflozin may prevent OSA (9). Based on prior findings (9), we hypothesized that ertugliflozin treatment would reduce the risk of new-onset OSA in the VERTIS CV trial. We found a significant 48% adjusted relative risk reduction that was observed with ertugliflozin versus placebo for incident OSA, similar to the magnitude of effect observed with empagliflozin [9].

Several potential mechanisms may underpin SGLT2i efficacy to improve OSA risk. The first is weight loss associated with SGLT2i, as urinary glucose excretion induced by this mechanism results in a daily net caloric loss. However, weight loss associated with SGLT2i is modest, and, in a mediation analysis from the EMPA-REG OUTCOME trial, weight loss was not significantly associated with the OSA effect [10]. It is also possible that the resultant reduction in plasma volume from osmotic diuresis (and accompanying natriuresis) decreases cardiac preload, resulting in less loop gain and downstream beneficial effects in respiratory physiology relevant to both obstructive and central sleep apnea (9).

There are several limitations to the present study. Patients were identified with OSA by investigator reporting of adverse events, which may have under-reported incident sleep apnea since periodic diagnostic sleep studies were not required in the protocol. Adverse event reporting is known to under-report incident OSA in large trials. Nonetheless, the results were highly consistent between the EMPA-REG OUTCOME trial and VERTIS CV, using two different SGLT2i. Furthermore, any misclassification of the outcome would tend to bias toward the null hypothesis and would not explain our observed results. The AHI was also not measured prospectively in this study; therefore, no comment can be made on the magnitude of AHI reduction or the baseline severity and type of sleep apnea in this population.

## Conclusion

In VERTIS CV, the SGLT2i ertugliflozin reduced the incidence of OSA in patients with T2D and ASCVD, an effect similar in magnitude to that previously reported with empagliflozin, contributing to the growing body of literature that SGLT2i may serve to prevent (and possibly treat) sleep apnea syndromes. Accordingly, the SGLT2i class deserves additional exploration for this potential new role, including in patients without diabetes, as well as a further inquiry into the mechanisms involved.

## Data Availability

Upon request, and subject to review, Pfizer will provide the data that support the findings of this study. Subject to certain criteria, conditions, and exceptions, Pfizer may also provide access to the related individual de-identified participant data. See https://www.pfizer.com/science/clinical-trials/trial-data-and-results for more information.
